# A Fast Deploying Monitoring and Real-Time Early Warning System for the Baige Landslide in Tibet, China

**DOI:** 10.3390/s20226619

**Published:** 2020-11-19

**Authors:** Yongbo Wu, Ruiqing Niu, Yi Wang, Tao Chen

**Affiliations:** Institute of Geophysics and Geomatics, China University of Geosciences, Wuhan 430074, China; wuarbo.309@gmail.com (Y.W.); taochen@cug.edu.cn (T.C.)

**Keywords:** fast monitoring, early warning, real-time, landslide

## Abstract

Landslide early warning systems (EWSs) have been widely used to reduce disaster losses. The effectiveness of a landslide EWS depends highly on the prediction methods, and it is difficult to correctly predict landslides in a timely manner. In this paper, we propose a real-time prediction method to provide real-time early warning of landslides by combining the Kalman filtering (KF), fast Fourier transform (FFT), and support vector machine (SVM) methods. We also designed a fast deploying monitoring system (FDMS) to monitor the displacement of landslides for real-time prediction. The FDMS can be quickly deployed compared to the existing system. This system also has high robustness due to the usage of the ad-hoc technique. The principle of this method is to extract the precursory features of the landslide from the surface displacement data obtained by the FDMS and, then, to train the KF-FFT-SVM model to make a prediction based on these precursory features. We applied this fast monitoring and real-time early warning system to the Baige landslide, Tibet, China. The results showed that the KF-FFT-SVM model was able to provide real-time early warning for the Baige landslide with high accuracy.

## 1. Introduction

Landslide hazard is one of the most common geological hazards in the natural world. They are also directly affected by human engineering activities. China is one of the countries most affected by landslide disasters in the world [1]. After the Ms 8.0 Wenchuan earthquake on 12 May 2008, tens of thousands of landslides over a broad area in west China were triggered. Some of these buried large areas of the town and dammed the rivers [2]. Thus, how to reduce property damage and casualties has always been an urgent problem. Landslide early warning systems (EWSs), which have already been working in many places of the world, were developed to fulfil this work [3,4,5,6,7].

According to the definition of the United Nations International Strategy for Disaster Reduction (UNISDR 2009), an EWS is defined as “the set of capacities needed to generate and disseminate timely and meaningful warning information to enable individuals, communities and organizations threatened by a hazard to prepare and to act appropriately and in sufficient time to reduce the possibility of harm or loss.” Referring to the above definitions, an efficient landslide EWS should comprise of four main sets of actions [8]: (1) monitoring activities, i.e., data acquisition, transmission, and maintenance of the instruments; (2) analysis and modelling of the phenomenon; (3) warning, i.e., the dissemination of simple and understandable information to the exposed elements; and (4) an effective response of the elements exposed to risk and the risk knowledge. Among the four sets of actions, (1) and (2) are critical for the successful early warning of an EWS.

In the regional scale landslide EWSs, a statistical method is used to determine the warning threshold. These landslide EWSs monitor the rainfall and make a classification early warning according to the rainfall threshold and the soil moisture [1,9,10,11,12,13,14]. Regarding a single landslide EWS, an early warning is made according to the induced factors and movement characters of the landslide [15]. Different instruments are used in single landslide EWSs to measure the induced factors and movement characters, for example, an inclinometer for tilt [16,17], fiber Bragg grating for fissures [18], an acoustic emission instrument for inner displacements [19,20], Ground-Based Synthetic-Aperture Radar, LiDAR(Light Detection and Ranging), total station, GPS and photogrammetric techniques for surface displacements [15,21,22,23,24], a geoelectrical monitor for soil moisture [25], and a wire extensometer for rock fracture [26]. These measuring data can be used to make early warnings with a single model or integrated models [27,28]. The majority of these models are derived from geological models. They usually make a classified early warning which is a qualitative analysis [18,26,27,28,29].

In this paper, we attempt to use the precursor features, specifically the vibration frequency, to carry out a quantitative analysis of landslide prediction. The Kalman filtering (KF)-fast Fourier transform (FFT)-support vector machine (SVM) model is proposed, which can build a grey box model for real-time early warning based on displacement data. The KF-FFT-SVM model uses Kalman filtering (KF) to make predications, fast Fourier transform (FFT) to extract precursor features, and the support vector machine (SVM) method to classify the precursor features. To ensure the KF-FFT-SVM model worked well, we fed it with real-time monitoring data. This required a stable and reliable monitoring system, which is especially important in the case of an emergency. In practice, most of the existing landslide monitoring systems are susceptible to breaking down in the field environment [30]. The measuring station was built with concrete which needs several days to harden, so it usually takes some more time to build a monitoring system, which therefore cannot immediately begin monitoring if a landslide suddenly occurred.

Thus, the FDMS was designed. It can increase the integration and reliability of the monitoring system and can be deployed immediately after a sudden landslide. In the FDMS, ad-hoc network technology is used to improve the robustness of the monitoring network and the measuring equipment is special designed to make it able to be installed quickly. The FDMS was applied in the Baige landslide, Tibet. In the FDMS, Beidou receivers, which are based on China’s Beidou Navigation Technology, and crack meters were used to monitor the displacements of the landslide. The KM-FFT-SVM model was trained by the displacement data, and the landslide precursor features were extracted successfully. Then, we used the testing data to verify the model. The results demonstrated that the KM-FFT-SVM model can make a real-time precursor predication with high accuracy and good practicability.

## 2. Fast Deploying Monitoring System

### 2.1. Traditional Monitor System

**Figure 1:**
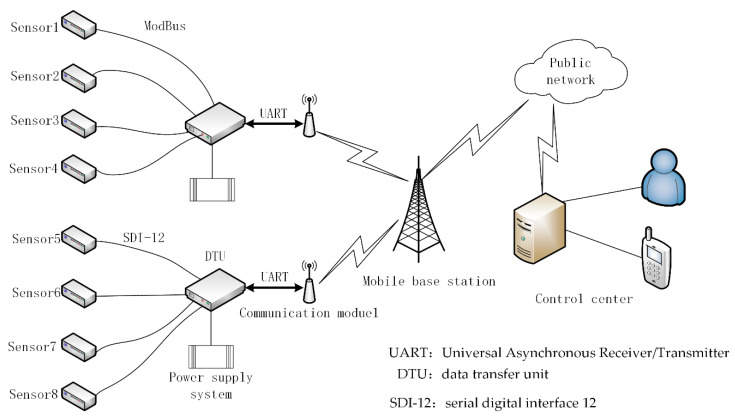
Traditional landslide monitoring system.

The structure of a traditional landslide monitoring system is shown in Figure 1. In Figure 1, all kinds of monitoring sensors are connected with the data transfer unit (DTU) by wires, and they communicate with each other in accordance with the Modbus protocol or SDI-12 protocol. The DTU, GPRS/3G/4G communication module, and power supply system constitute a remote measurement unit (RTU). The monitoring data are sent to the mobile communication network by the communication module and transmitted to the control center through the public network. In this way, the system robustness is poor. If the GPRS/3G/4G communication module of one monitoring point breaks down, all the data from the sensors under this monitoring point will not be submitted, which means a partial paralysis of the monitor system. Therefore, a more flexible and stable networking structure is required to improve the robustness of the traditional landslide monitoring system. This is the adaptive landslide monitoring system.

### 2.2. Composition of FDMS

**Figure 2:**
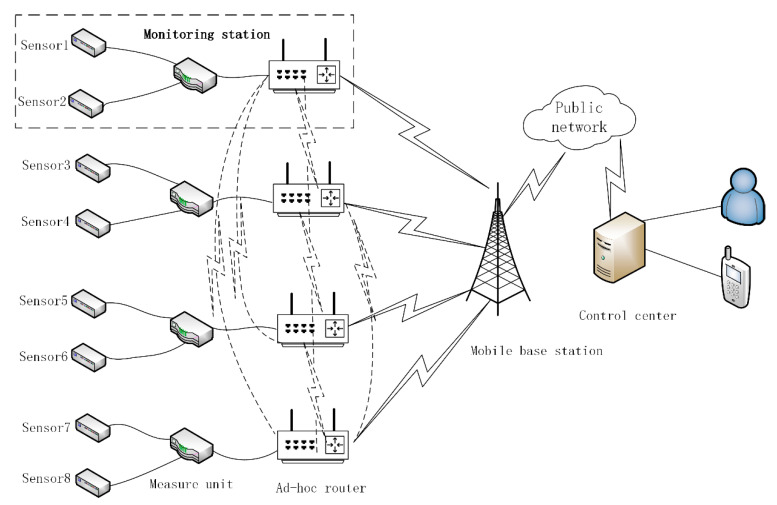
Fast deploying monitoring system.

**Figure 3:**
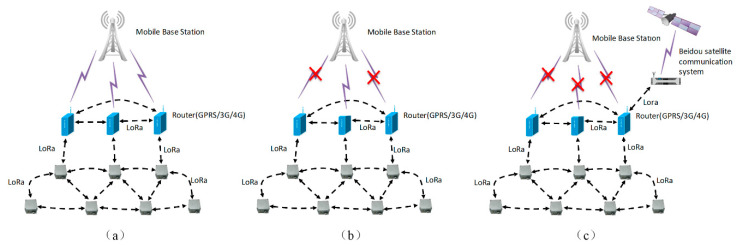
Working mode. Normal mode (**a**), Communication fault mode (**b**), and Beidou satellite communication mode (**c**).

An FDMS is an adaptive landslide monitoring system that is based on an ad-hoc network. An ad-hoc network is more secure, robust, stable, and reliable when compared with traditional bus and star networks. Figure 2 is the typical structure of a FDMS. In Figure 2, each station is composed of several sensors, a data acquisition instrument, and an ad-hoc router. The ad-hoc routers have GPRS/3G/4G communication modules and form a wireless local ad-hoc network using LoRa technology. They communicate with each other by multi-hops and can also act as access point (AP) nodes that have the ability to gain access to the external network. In the wireless local ad-hoc network, when one node breaks down, the network will find new paths to skip this node through a routing algorithm, which improves the network’s robustness. The FDMS has three working modes. One is the Normal mode as shown in Figure 3a; another is the Communication fault mode as shown in Figure 3b. In this mode, if the GPRS/3G/4G communication module in some of the ad-hoc routers breaks down, the system finds a new routing path to send the data. The third is the Beidou satellite communication mode as shown in Figure 3c. This mode means that if the GPRS/3G/4G communication modules in all of the ad-hoc routers break down, then the Beidou satellite communication system will be started. With the advantages mentioned above, the FDMS is highly robust and works well in field environments, especially in places where there are no mobile signals, or the signals are weak. Furthermore, the measuring equipment is specially designed to make it deployed easily and quickly, as described in more detail below.

### 2.3. FDMS in Baige Landslide

**Figure 4:**
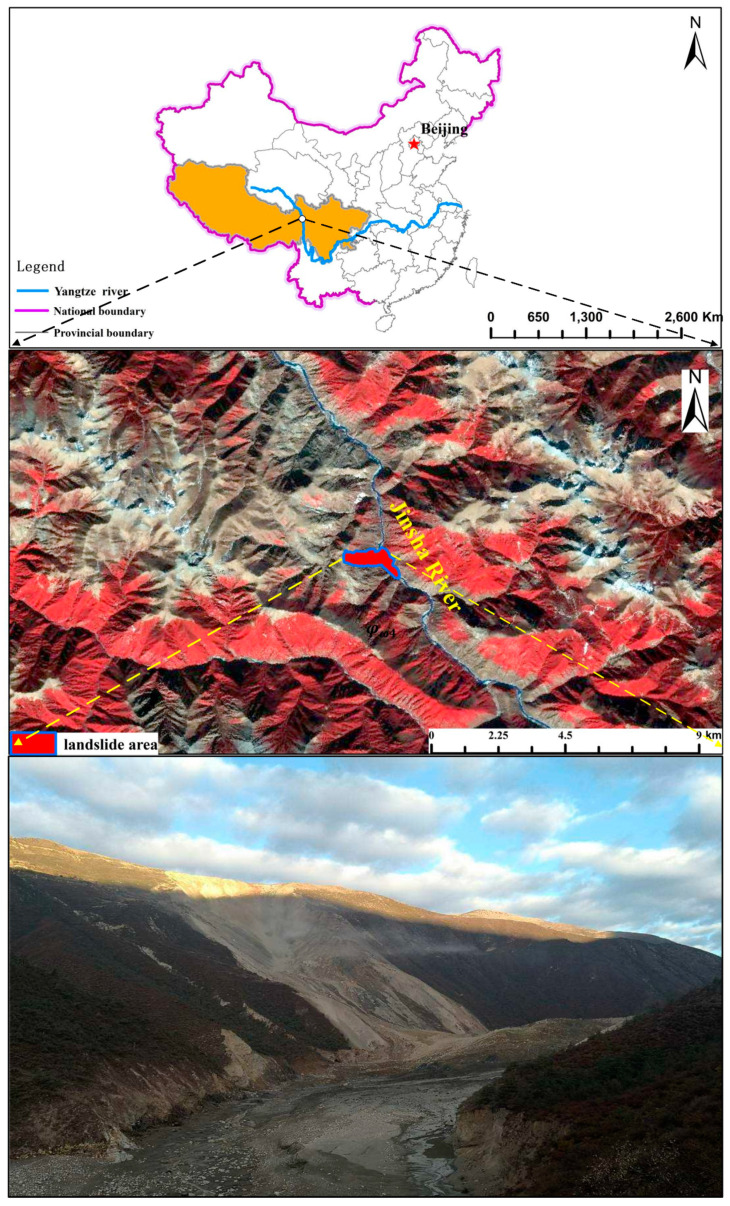
Location of the Baige landslide.

**Figure 5:**
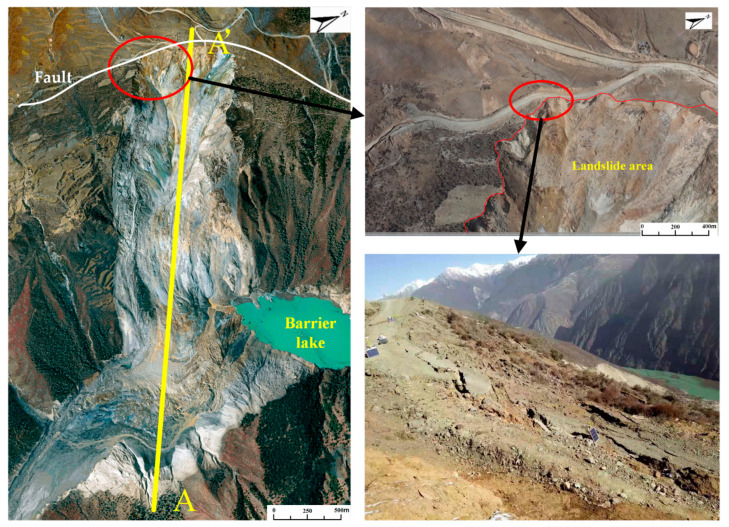
Photograph of the Baige landslide and details of the sliding surface.

**Figure 6:**
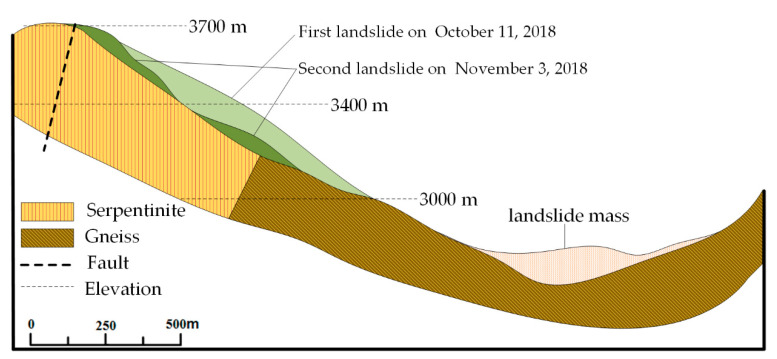
Geological cross-section of Baige landslide (line A’-A).

In the early morning of 11 October 2018, a large scale landslide occurred on the Tibetan Bank of the Jinsha River at the junction of Baige Village, Boro Township, Jiangda County, Tibet Autonomous Region and Zeba Village, Ronggai Township, Baiyu County, Sichuan Province. The landslide blocked the Jinsha River and formed a barrier lake. Then, late on November 3, a second landslide occurred and blocked the Jinsha River again. The location of the landslide is shown in Figure 4. Figure 5 shows a photograph of the Baige landslide and the details of the sliding surface. In Figure 5, a fault extending along the road at the back of the landslide can be seen. On the top of the landslide, the white/green cataclastic serpentine is exposed, and its thickness is about 300 m. The serpentine is mainly located in the up area of the landslide, between the elevations of 3400–3700 m. Below 3400 m, Gneiss is exposed (Figure 6). The dip direction and the dip are 235° and 40°, respectively. There are two structural planes in the side of the landslide. The dip direction and the dip of one plane are from 60° to 80° and from 75° to 85°, respectively. The other are 100° to 115° and 80°. Because of the two structural planes, which formed during the landslide, a wedge-shaped groove which is thick in the middle and thin in the sides was formed. The average slope length of the landslide was about 790 m and the width was about 500 m. The average thickness of the landslide was about 50 m and the maximum thickness may have been 80 m (Figure 5). In the first landslide, there were approximately 2.2 × 10^7^ m^3^ of rock and soil that fell into the Jinsha River and built a dam in the valley. Then, a barrier lake was formed by the dam (Figure 5). The length of the barrier lake was about 1100 m, and the width was about 500 m. The maximum height of the dam over the original river surface was about 85 m. The second landslide occurred at the back edge of the first landslide. In this case, the total volume of the rocks falling down were about 8.5 × 10^6^ m^3^. The unstable rock mass scraped the broken rock mass along the way, which added 0.8 × 10^6^ m^3^ more rock falling down. The rocks blocked up the Jinsha River again and increased the height of the dam formed in the first landslide by 50 m [31]. The barrier lake formed by the two landslides was a great threat to the people living in the lower reaches of the Jinsha River.

**Figure 7:**
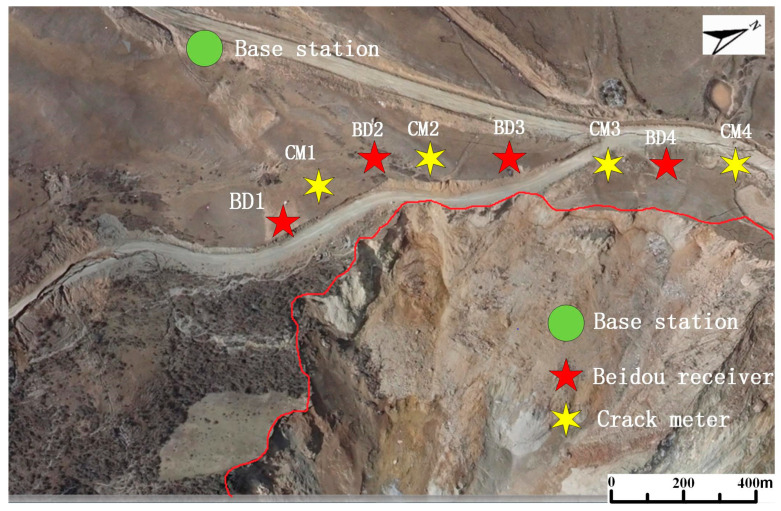
Locations of the equipment on the landslide.

The Baige landslide occurred suddenly. There were no monitoring devices working there previously. There were also no mobile signals. A monitoring system needed to be set up immediately to ensure the safety of the emergency rescue workers dredging the barrier lake. Thus, the FDMS was applied there. The locations of the monitoring equipment are shown in Figure 7. BD1, BD2, BD3, and BD4 represent the Beidou receivers, while CM1, CM2, CM3, and CM4 represent the crack meters. The equipment was located in the back edge of the slope to monitor the movements of the landslide. In this FDMS, the surface displacements were measured by Beidou receivers and the crack widths by the crack meters. The sampling time step was 10 min.

**Figure 8:**
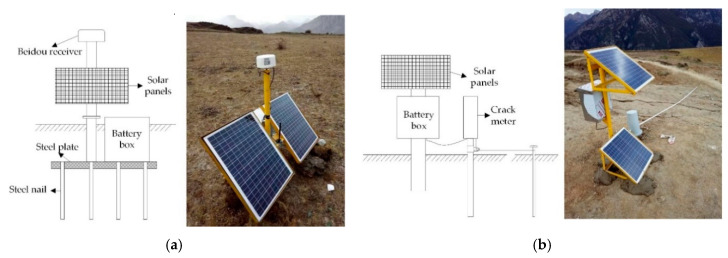
Monitoring equipment on the landslide. (**a**) Beidou receiver. (**b**) Crack meter.

The Beidou receivers were Northdoo global navigation satellite system (GNSS) devices. Their plane accuracy is (8 + 1 ppm) mm, and the elevation accuracy is (15 + 1 ppm) mm. This is much better than the differential global position system (DGPS) working model, which has a plane accuracy of (3 + 1 ppm) mm, and an elevation accuracy of (5 + 1 ppm) mm. The differential working model requires a fixed base station on the stable rocks 1 km away from the moving Beidou receivers, as shown in Figure 7. The installation of the Beidou receivers is shown on the left side of Figure 8a.

The traditional method is to fix the supporting stake with concrete, but it takes several days for the concrete to harden. Here, no concrete was used. The Beidou receiver supporting stake was fixed on a steel plate, which was nailed to the bottom of a 1 m deep hole. To make the foundation firmer, the metal battery box was put on the steel plate as a counterweight. This installed method is time saving comparing to the traditional method. The Beidou receiver is designed in accordance with the IP67 (ingress protection rating 67). It is dust-proof, rain-proof, and snow-proof.

The installation of the crack meter is shown on the left side of Figure 8b. The crack meter was connected to a metal pole which was buried 1 m deep in the slope at one side of the ground crack. A pull wire drawn from the crack meter was fixed on the metal pole at the other side of the ground crack. The pull wire could extend as the ground crack expands, and so the width of the crack can be determined by measuring the change of the pull wire. The measuring range of the crack meter was 5 m, and the accuracy was 0.5 mm.

## 3. Early Warning Model

### 3.1. Kalman Filtering

Kalman filtering (KF), which was put forward by R.E. Kalman in 1960, is a linear recursive filtering method based on linear unbiased minimum variance estimation theory. It combines the concept of state space with filtering theory. The cores of this method are the transition equation and the recursive formula. With them, new state and observation values can be predicted according to the estimated value at a previous moment and the observation value at the present moment.

Given a discrete time system, $X_{1}$, $X_{2}$, $X_{3},\cdots$,$X_{k}$ are the system state vectors at $kT_{s}$, where $X_{k}\in R^{n}$, $T_{s}$ is the measuring interval of the system. We defined $U_{k}$ as the system control input vector and $W_{k}$ as incentive noise vector, and the state transition equation is described in Equation (1).

(1) $$X_{k}=AX_{k-1}+BU_{k}+W_{k}$$

Defining $Z_{k}\in R^{n}$ as the observation vector and $V_{k}$ as the observation noise vector, we obtained the observation formula:

(2) $$Z_{k}=HX_{k}+V_{k}.$$

*A*, *B*, *H* is the state transition matrix, and $W_{k}$, $V_{k}$ are the independent and normal distribution of white noise:

(3) $$W_{k}\sim N\left(0,Q\right).$$

(4) $$V_{k}\sim N\left(0,R\right).$$

In the state estimating of a discrete time system, Formula (1) is used to obtain ${\hat{X}}_{j|k}$, which is the best estimating value of $X_{j}$ at $T_{s}$, and there are three situations:

(a) When j = k, ${\hat{X}}_{j|k}$ is the optimum filtering of $X_{k}$.
(b) When j > k, ${\hat{X}}_{j|k}$ is the optimum predicting of $X_{k}$.
(c) When j < k, ${\hat{X}}_{j|k}$ is the optimum smoothing of $X_{k}$.

The recursive formula can be described as time update process and state update process. The time update process:

(5) $${\hat{X}}_{k|k-1}=A{\hat{X}}_{k-1|k-1}+BU_{k-1}$$

(6) $$P_{k|k-1}=AP_{k-1|k-1}A^{T}+Q$$

where *P* is the error estimating covariance matrix:

(7) $$E_{k}=X_{k}-{\hat{X}}_{k}$$

(8) $$P_{k}=E\left(E_{k}E_{k}^{T}\right).$$

The state update process:

(9) $$K_{k}=P_{k|k-1}H^{T}{\left(HP_{k|k-1}H^{T}+R\right)}^{-1}$$

(10) $${\hat{X}}_{k|k}={\hat{X}}_{k|k-1}+K_{k}\left(Z_{k}-H{\hat{X}}_{k|k-1}\right),$$

(11) $$P_{k|k}=\left(I-K_{k}H\right)P_{k|k-1}$$

**Figure 9:**
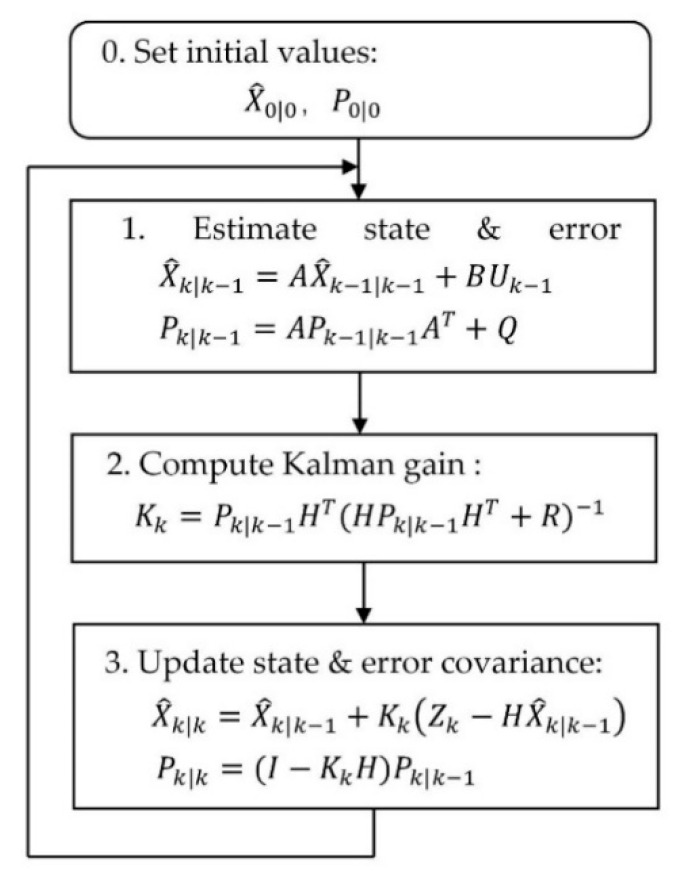
The computational process of the Kalman filtering (KF) algorithm.

The computational process of the KF algorithm is shown in Figure 9.

### 3.2. Fast Fourier Transform

Fast Fourier transform (FFT) is an efficient algorithm for a discrete Fourier transform (DFT). Given the finite sequence length x(n) with length N, the DFT is described as:

(12) $$X\left(k\right)=\overset{N-1}{\underset{x=0}{\sum }}x\left(n\right)W_{N}^{\mathrm{nk}}$$

FFT uses the symmetry, periodicity, and reducibility characters of $W_{N}^{nk}$, i.e., Equations (13)–(15), to decompose a large scale DFT into a combination of several small scale DFTs.

(13) $${\left(W_{N}^{\mathrm{nk}}\right)}^{*}=W_{N}^{-\mathrm{nk}}=W_{N}^{\left(N-n\right)k}=W_{N}^{n\left(N-k\right)},$$

(14) $$W_{N}^{\mathrm{nk}}=W_{N}^{\left(N+n\right)k}=W_{N}^{n\left(N-k\right)}$$

(15) $$W_{N}^{\mathrm{nk}}=W_{\mathrm{mN}}^{\mathrm{mnk}}=W_{N/m}^{\mathrm{nk}/m}$$

The following is a time-based 2-FFT algorithm. Given N = 2M, then x (n) can be divided into two groups. When n is even, let n = 2r. When n is odd, let n = 2r+1. Let x (2r) = x_1_(r), X_1_ (k)= DFT [x_1_ (r)], x (2r+1) = x_2_ (r), X_2_ (k) = DFT [x_2_ (r)], where r = 0, 1,…, N − 1. Then Formula (12) can be rewritten as:

(16) $$X\left(k\right)=X_{1}\left(k\right)+W_{N}^{k}X_{2}\left(k\right),$$

(17) $$X\left(k+N/2\right)=X_{1}\left(k\right)+W_{N}^{k}X_{2}\left(k\right).$$

We can calculate that an N-point FFT operation needs (N/2) log_2_N complex multiplication and Nlog_2_N complex addition, which greatly improves the operation efficiency of DFT.

### 3.3. Support Vector Machine

A support vector machine (SVM) is a supervised machine learning technique for constructing the optimal hyperplane based on the principle of structural risk minimization. It maps the input vectors into high dimensional feature space by non-linear transformation and finds the optimal classification hyperplane in high dimensional feature space, which separates the data into two groups with a maximized classification interval. Suppose a training sequence $\left\{x_{i},y_{i}\right\}$, *i* = 1, 2, …, *l*; $x_{i}\in R^{n}$, $y_{i}\in \left\{-1,+1\right\}$; where l is the number of samples, and n is the dimension of $x_{i}$. In the case of linearly separable values, a classification hyperplane w+b=0 can be found to separate $\left\{x_{i},y_{i}\right\}$ into two groups. For the nonlinearity situation, $x_{i}$ is mapped from a low dimension feature space to a high dimension feature space by a nonlinear mapping function Φ(x). Then, the classification hyperplane can be expressed as wΦ(x)+b=0, where w, *b* are constants to be determined. Finding the classification hyperplane equals maximizing 2/‖w‖. This problem can be solved by the Lagrange multiplier method:

(18) $$\{\begin{array}{c} \min\frac{{\left\|w\right\|}^{2}}{2}+C\overset{l}{\underset{i=1}{\sum }}\xi_{i},\\ s.t.\;y_{i}\left(w\cdot x_{i}+b\right)\geq 1-\xi_{i},\\ \xi_{i}\geq 0,i=1,2\ldots ,l \end{array}$$

where $\xi_{i}$ is the relaxation factor, and C is the penalty factor. The dual problem is given by the Karush–Kuhn–Tucher (KKT) conditions:

(19) $$\left\{ \begin{array}{c} \max\overset{l}{\underset{i=1}{\sum }}\alpha_{i}-\frac{1}{2}\overset{l}{\underset{i=1}{\sum }}\overset{l}{\underset{j=1}{\sum }}\alpha_{i}\alpha_{j}y_{i}y_{j}\varphi \left(x_{i}\right)\cdot \varphi \left(x_{j}\right)\\ s.t.\;0\leq \alpha_{i}\leq C,\overset{l}{\underset{i=1}{\sum }}\alpha_{i}y_{i}=0. \end{array}\right.$$

Solving the problem using the sequential minimal optimization (SMO) algorithm, we can obtain the classification function:

(20) $$f\left(x\right)=\mathrm{sign}\left\{\overset{l}{\underset{i=1}{\sum }}\alpha_{i}y_{i}\left[\Phi \left(x_{i}\right)\cdot \Phi \left(x_{i}\right)\right]+b\right\}$$

If the kernel function $K\left(x_{i},x_{i}\right)=\Phi \left(x_{i}\right)\cdot \Phi \left(x_{i}\right)$ is found here, the function operation in Equation (20) can be simplified to the inner product of the vectors. The commonly used kernels are the linear kernel function, polynomial kernel function, radial basis (Gauss) function, and sigmoid kernel function.

### 3.4. The Proposed KF-FFT-SVM Model

**Figure 10:**
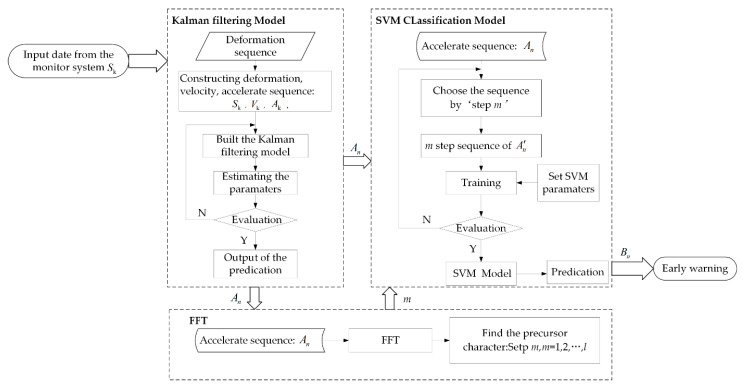
Process of the Kalman filtering (KF)-fast Fourier transform (FFT)-support vector machine (SVM) model.

A landslide can be treated as a multi-dimensional nonlinear dynamic system influenced by various factors [32]. In practice, it is difficult to simulate a nonlinear dynamic system; however, we can treat it as a linear model in a short time period. Therefore, we proposed the KF-FFT-SVM model. This model can make a real-time prediction by analyzing and extracting the precursor features from the landslide displacement data in a certain period of time. The whole process of the KF-FFT-SVM model is shown in Figure 10. Firstly, we put $X_{k}=\left(S_{k},V_{k},A_{k}\right)$, where $V_{k}$ and $A_{k}$ are the velocity and acceleration of the landslide surface displacement data sequence $S_{k}$ obtained by the Beidou receivers, into the Kalman filtering to obtain the prediction result of $A_{n}$. Formula (21) is the prediction precision evaluation of Kalman filtering. Secondly, we used FFT to analyze the spectrum characteristics of $A_{n}$ near the occurring time of the landslide and found the ‘step length m’, which represents the precursor vibration period of the landslide slope failure. Finally, $A_{n}^{'}=[A_{n},A_{n+1},\ldots ,A_{n+m-1},label]$, which represents the training data and testing data which were generated from $A_{n}$ according to the precursor character ‘step length m’. Then, the SVM model was trained by the labelled training data, and the trained SVM model could be used to make the real-time prediction. The prediction result $B_{n}$ is a vector with the same dimension of $A_{n}$ and its value is either ‘−1′or ‘1′. ‘−1′ represents that there is no warning, while ‘1′ represent that there is a warning. The classification accuracy of SVM is given in Formula (22). The proof of the mathematical new relationships is in Appendix A.

(21) $$\mathrm{RMS}=\frac{1}{M}\sqrt{\overset{N}{\underset{i=1}{\sum }}{\left(X_{i}-Z_{i}\right)}^{2}}$$

(22) $$Accuracy=\frac{Right\;classification\;numbers}{whole\;samples}$$

## 4. Real-Time Prediction

### 4.1. Data Pre-Processing

**Figure 11:**
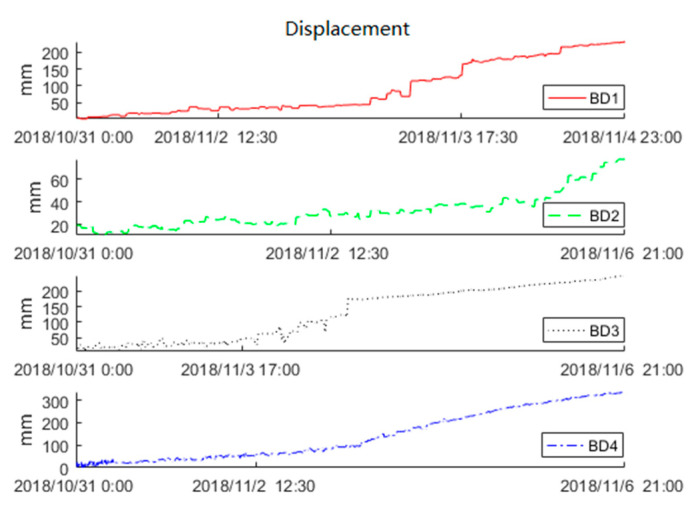
Raw displacement data.

**Figure 12:**
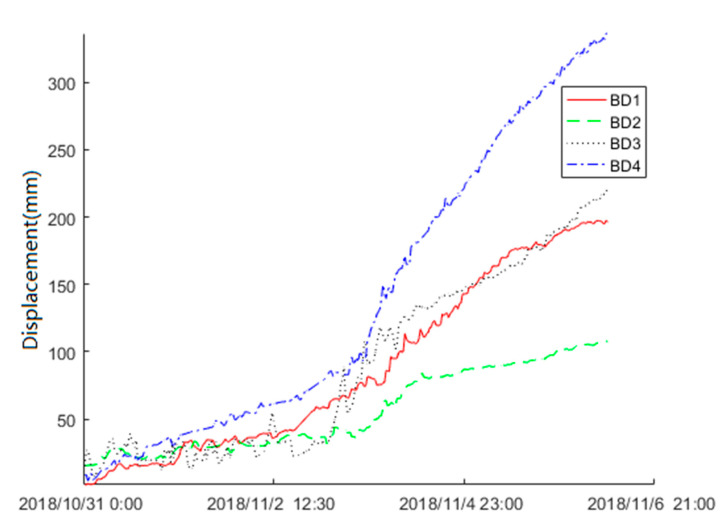
Sampling of the raw displacement data.

The surface displacement data of the Baige landslide was recorded by the FDMS starting on 31 October 2018. On late 3 November, the second landslide occurred. To find the precursor feature, we used the displacement data obtained by Beidou receivers from 31 October to 6 November to train the KF-FFT-SVM model. Figure 11 shows the raw data of BD1, BD2, BD3, and BD4. The Beidou receiver recorded the three-dimensional (X, Y, Z) displacements of the landslide, while, for simplicity, only the horizontal (X) displacement data were used here. The raw displacement data were obtained at an interval of 10 min. From Figure 11, there were no displacements within 30 min for the majority of the time. Therefore, we sampled the raw data at intervals of 30 min. The sampling result is shown in Figure 12, and this was used to train the KF-FFT-SVM model instead of the raw data.

### 4.2. KF-FFT-SVM Model Building

#### 4.2.1. KF Predicting

To build the KF model of the displacement data, we chose the displacement, displacement velocity, and displacement acceleration as the state vectors, which are *S*_k_, *V_k_*, and *A_k_*, respectively. The relations between them are:

(23) $$\{\begin{array}{c} X_{k}={\left[S_{k},V_{k},A_{k}\right]}^{T},\\ S_{k}=S_{k-1}+V_{k-1}\cdot T_{s}+w_{k-1}^{1},\\ V_{k}=V_{k-1}+A_{k-1}\cdot T_{s}+w_{k-1}^{2},\\ A_{k}=A_{k-1}+w_{k-1}^{3} \end{array}$$

where $T_{s}$ is the data sampling interval, and $w_{k}^{1}$, $w_{k}^{2}$, and $w_{k}^{3}$ are the random errors. Let $T_{s}=1$, then the stochastic difference equation of the system state is:

(24) $$X_{k}=\left[\begin{array}{ccc} 1 & 1 & 0\\ 0 & 1 & 0\\ 0 & 0 & 1 \end{array}\right]X_{k-1}+[w_{k-1}^{1},w_{k-1}^{2},w_{k-1}^{3}{]}^{T}$$

**Figure 13:**
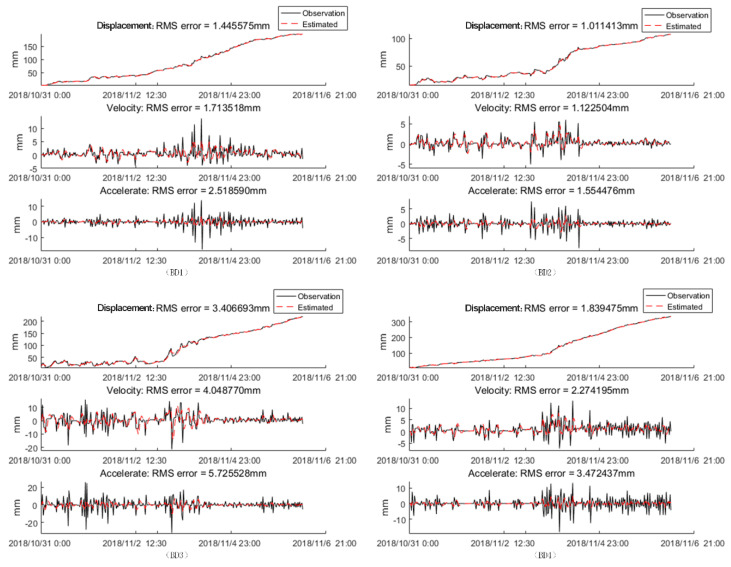
The Kalman filtering results.

The observation formula is described as Formula (25):

(25) $$Z_{k}=\left[1\;1\;0\right]X_{k-1}+v_{k-1}$$

where $v_{k}$ is the random error, $A=\left[\begin{array}{ccc} 1 & 1 & 0\\ 0 & 1 & 0\\ 0 & 0 & 1 \end{array}\right]$ and H = [1,1,0]. The random errors $w_{k}$ and $v_{k}$ are unknown, and our purpose was to use Formulas (5)–(11) to determine them. To solve this problem, we set *Q* and *R* with random values, and used the pre-processing data in Section 4.1 to find the values of *Q* and *R* that made the KF model converge to the optimal solution. Figure 13 shows the estimated result of BD1, BD2, BD3, and BD4, which are the red curves. In this KF model, Q = [5,3,3]*^T^*, and R = 3. The maximum estimated error is 5.73 mm, which indicates that the KF model we built had a good prediction and filtering result.

#### 4.2.2. FFT Analysis

**Figure 14:**
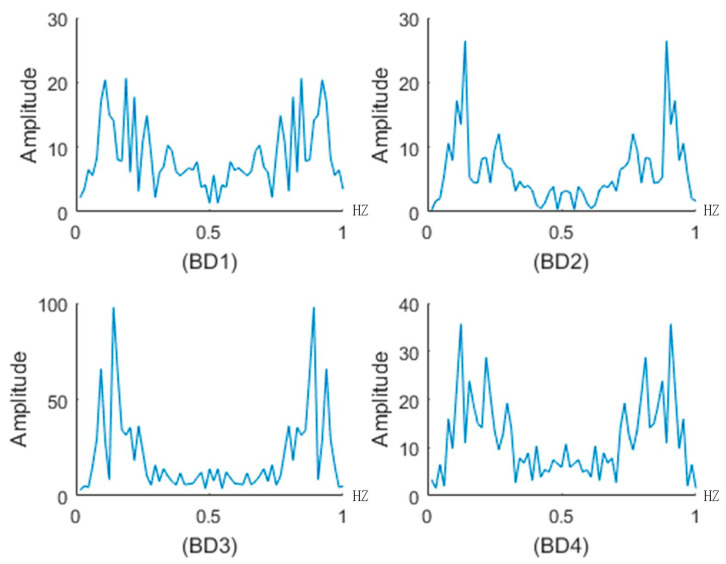
FFT of the displacement acceleration data in the secondary landslide.

In the FFT analysis, we chose 64 displacement acceleration data values between 3 November and 4 November to conduct FFT, because during this period the secondary landslide happened. Figure 14 shows the FFT result. The FFT length N was 64, and the data sampling interval was $T_{s}$. Let $T_{s}=1$, and the sampling frequency can be simplified to 1 Hz. In Figure 14, there are two major amplitude peak values near 0.2 Hz and 0.9 Hz, which indicate that the precursor vibration period of the landslide was approximately 5$T_{s}$ in the time domain. We chose the step length m=2,3,4,5,6,7,8 to construct the displacement acceleration sequence.

#### 4.2.3. SVM Model Training

Before the SVM model was trained, we marked the displacement acceleration data of BD1, BD2, and BD3 manually. The data between 3 November and 4 November were marked with the label “Positive”, which signified that there was an early warning, and the others were marked with the label “Negative”, which signified that there was no early warning. Then, we use the marked BD1, BD2, and BD3 data to construct the displacement acceleration sequence $A_{n}^{\prime }$,

(26) $$A_{n}^{'}=[A_{n},A_{n+1},\ldots ,A_{n+m-1},label]$$

where m=2,3,4,5,6,7,8, which is given in part 4.2.2; n=1,2,…,336−m; and *label* is the marked value of $A_{n}$.

Formula (27) is an example of data set when *m* = 2. Here, $A_{n\left(BD1\right)}^{'}$, $A_{n\left(BD2\right)}^{'}$, and $A_{n\left(BD3\right)}^{'}$ were chosen as training data. We also constructed $A_{n\left(BD4\right)}^{'}$ as testing data with the same method.

(27) $$\{\begin{array}{c} A_{n\left(BD1\right)}^{'}={\left[A_{n},A_{n+1},\ldots ,A_{n+m-1},label\right]}_{\left(BD1\right)}\\ training\;data:A_{n\left(BD2\right)}^{'}={\left[A_{n},A_{n+1},\ldots ,A_{n+m-1},label\right]}_{\left(BD2\right)}\\ A_{n\left(BD3\right)}^{'}={\left[A_{n},A_{n+1},\ldots ,A_{n+m-1},label\right]}_{\left(BD3\right)}\\ testing\;data:\;A_{n\left(BD4\right)}^{'}={\left[A_{n},A_{n+1},\ldots ,A_{n+m-1},label\right]}_{\left(BD4\right)} \end{array}$$

**Figure 15:**
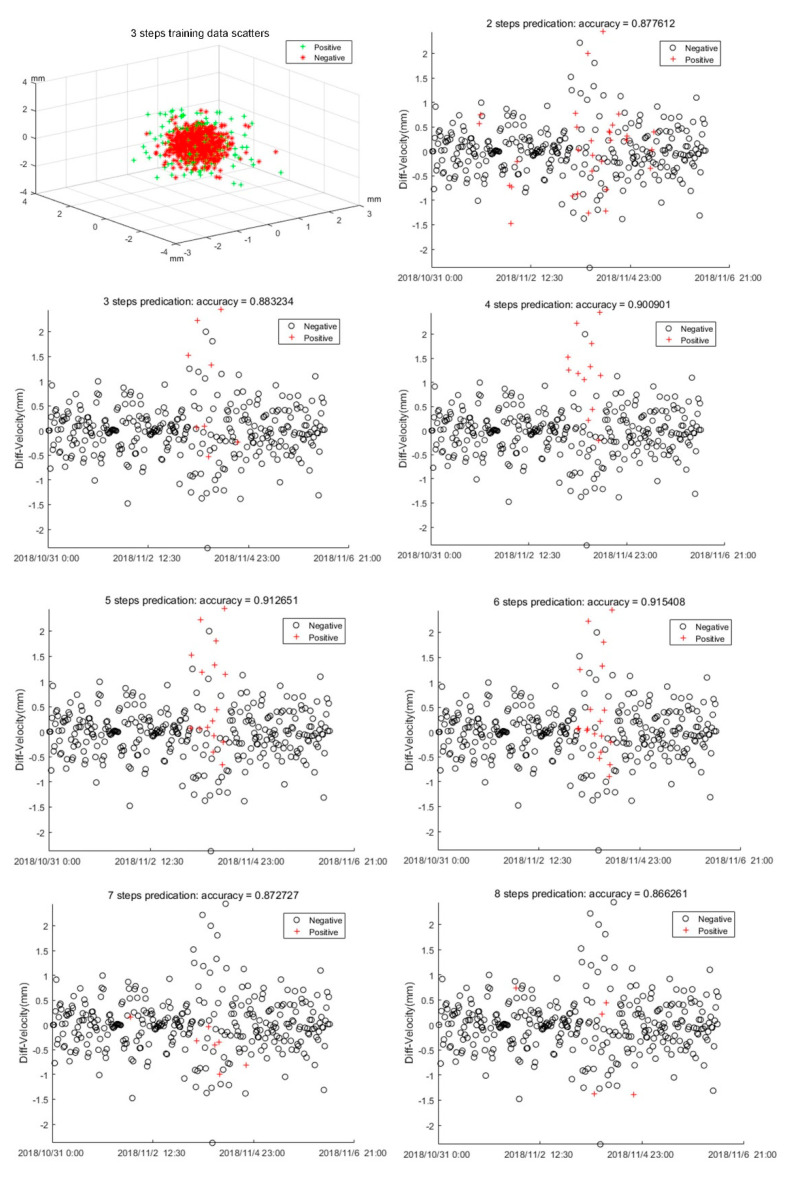
Prediction results at different step length sequences.

In SVM training, the radial basis function (RBF) function and SMO algorithm are used to search for the best *C* and γ. The early warning prediction results of the testing data $A_{n\left(BD4\right)}^{'}$ with different step sequences are shown in Figure 15. Figure 15 also shows the three step length training data scattered in 3D space, and it is clear that they cannot be well-separated in 3D space. Therefore, we separated them in a higher dimension. From Figure 15, we know that when *m* = 6, the optimal result is achieved. In this situation, the highest accuracy = 0.915, *C* = 4, and γ=1. The result is in great agreement with the FFT analysis, which shows the best precursor landslide character is near 0.2 Hz, which is equal to six step lengths in the time domain.

### 4.3. Application of the Real-Time Prediction Method

The real-time prediction method based on the KF-FFT-SVM model makes landslide predictions according to the principle that the mechanical vibrations of the landslide slope failure are recorded in the displacement data. In this study, the Beidou receivers had the ability to measure the displacement of the landslide at the frequency of every 10 min with an accuracy of 3 mm, which was given by the manufacturer. The raw displacement data are shown in Figure 11, and it is clear that there are random errors from the Beidou receivers as the displacement curves are not continuous in their rise in several periods. Thus, the raw data were pre-processed, and the 30 min statistics of the raw displacement data are given in Figure 12. Supposing 30 min as the unit time, then the KF method was used to filter the random error and to make a prediction of the displacement for the next 30 min. FFT, here, was used to find the precursory vibration frequency of the displacement acceleration before the landslide slope failure. When the precursory vibration frequency was found, we could generate the training data from the BD1, BD2, and BD3 and train the SVM by these data. The trained SVM was tested by the data obtained by BD4 as shown in Figure 15.

The trained KF-FFT-SVM model can make online predictions. The displacement data obtained by the FDMS acts as the input for the KF-FFT-SVM model. The outcome of the KF-FFT-SVM model is the precursory warning of a landslide. If the outcome of the KF-FFT-SVM model is “Positive”, the early warning will be triggered. However, if the outcome is “Negative”, there will be no early warning. In this study, the sample period of the data was 30 min; therefore, the KF could predict the landslide displacement in the next 30 min. This indicates that the KF-FFT-SVM model can forecast landslide slope failure at least 30 min in advance. In practice, the parameters of KF-FFT-SVM model are pre-set; however, the model can be retrained, and the parameters can be adjusted dynamically.

## 5. Discussion

Landslide EWSs are widely used to reduce disaster losses. The effectiveness of a landslide EWS depends on the reliability of monitoring and accurate early warnings. In this paper, we focused on the monitoring and warning methods of a landslide EWS. Effective responses after the landslide are beyond the discussion of this paper. To improve the robustness of the monitoring system of a landslide EWS, we designed the FDMS. With the real-time monitoring landslide displacement data obtained by the FDMS, we used the KF-FFT-SVM model to predict landslide slope failure with high accuracy. The prediction method we proposed here could forecast the landslide in advance, and is a timely online prediction method.

Although the real-time prediction method proposed here showed a good result in the Baige landslide, there are still limitations in practice. The precursory features of different landslides may be different, and thus the trained SVM model cannot be effective for all type of landslides. The SVM model should be trained by the critical slide data of the landslide; however these critical slide data are difficult to obtain as there may be no monitoring equipment working wherever a landslide occurs, or if the equipment is destroyed after the landslide slope failure.

In this study, we were lucky to obtain the critical slide data to train the KF-FFT-SVM model. To make the model effective for more landslides, we should use as many historical critical slide data as possible to train the model and adjust the model parameters for different types of landslides. There are also uncertainties in the application of the real-time prediction method. The most important feature of this method is that it uses the surface displacement data to find the precursory features. The problem is that, in the KF-FFT-SVM model, we consider the surface displacement data measured by the Beidou receivers as the mechanical vibration of the landslide, but there must be distortions when the vibration signals are transmitted to displacement signals.

The method proposed in this study provides a new idea for the real-time early warning of landslides. In this method, a landslide is considered as a quadratic dynamical system, and we extracted the precursory features from the surface displacement data of the landslide and used machine learning to construct the early warning model. By introducing the concept of quadratic dynamical systems and machine learning, the real-time prediction method makes the landslide early warning easier, and the only thing needed is to train the model with plenty of data.

The real-time prediction method is also practical and has strong promotion value. The majority of the landslide EWSs in the literature have very complex monitoring systems and make an early warning simply by a pre-set threshold, which produces a lack of accuracy and timeliness [27,28]. In the real-time prediction method, all the displacement data obtained by different instruments can be used to extract precursory features and train the KF-FFT-SVM model. In this study, we only used the surface displacement data, and this method would be even more effective if the deep-seated information of the landslide is used.

## 6. Conclusions

In conclusion, the monitoring and early warning method proposed in this paper improved the effectiveness of the landslide EWS. This FDMS is simple, low-cost, and has high robustness. The real-time prediction method made the landside early warning more accurate and timelier by extracting the precursor features, which are considered as the inner mechanical vibrations of the landslide slope failure, from the landslide surface displacement data.

The key to the real-time prediction method lies in correctly extracting the inner vibrations of the landslide. In future studies, we will perform research on the relationship between the surface displacements and inner vibration of the landslide, which is measured by rock or soil press sensors. We will also study the method of extracting precursory features from 2D and 3D surface and deep-seated displacement data.

## Appendix A

### Proof of Mathematical New Relationships

In the KF-FFT-SVM model, the KF algorithm is used for predicting $A_{k}$, which is the acceleration of landslide displacement. $A_{k}$ represents the high frequency component of the landslide displacement, which may contain more useful feature information. This is why we chose $A_{k}$ as an analysis object. The prediction of $A_{k}$ is expressed as $A_{n}$ here. The FFT algorithm is used for analyzing and extracting the feature information in $A_{n}$. In this study, the feature information was the vibration frequency indicated in $A_{n}$. If the characteristic frequency is *f*, then we construct the sequence $A_{n}^{'}=[A_{n},A_{n+1},\ldots ,A_{n+m-1},label]$ (*m* is an integer here, *m* = 1/*f*), which represents the one period characteristic waveform. Then, we used $A_{n}^{'}$ to train the SVM model. However, 1/*f* is not always an integer. In this situation, given that the approximation of *m* is *p*, then the sequences should be constructed like this:

(A1) $$\left\{ \begin{array}{c} A_{n}^{'}\left(p-q-1\right)=[A_{n},A_{n+1},\ldots ,A_{n+p-q-1},label]\\ A_{n}^{'}\left(p-q\right)=[A_{n},A_{n+1},\ldots ,A_{n+p-q},label]\\ \vdots \\ A_{n}^{'}\left(p-1\right)=[A_{n},A_{n+1},\ldots ,A_{n+p-1},label]\\ \vdots \\ A_{n}^{'}\left(p+q-2\right)=[A_{n},A_{n+1},\ldots ,A_{n+p+q-2},label]\\ A_{n}^{'}\left(p+q-1\right)=[A_{n},A_{n+1},\ldots ,A_{n+p+q-1},label] \end{array}\right.$$

Here, *q* is an integer, 0≤q≤p−2. Then, we used the sequence list in Formula (A1) one by one to train the SVM and find the best fitting one as the feature ‘step length’.

In this study, the characteristic frequency *f =* 0.2 and 0.9. Thus, we chose the step length m=2,3,4,5,6,7,8 and constructed sequences like this:

(A2) $$\left\{ \begin{array}{c} A_{n}^{'}\left(2\right)=[A_{n},A_{n+1},label]\\ A_{n}^{'}\left(4\right)=[A_{n},A_{n+1},A_{n+2},label]\\ A_{n}^{'}\left(4\right)=[A_{n},A_{n+1},\ldots ,A_{n+3},label]\\ A_{n}^{'}\left(5\right)=[A_{n},A_{n+1},\ldots ,A_{n+4},label]\\ A_{n}^{'}\left(7\right)=[A_{n},A_{n+1},\ldots ,A_{n+5},label]\\ A_{n}^{'}\left(7\right)=[A_{n},A_{n+1},\ldots ,A_{n+6},label]\\ A_{n}^{'}\left(8\right)=[A_{n},A_{n+1},\ldots ,A_{n+7},label] \end{array}\right.$$

We trained the SVM with these eight sequences, respectively, with the method in Section 4.3, and found the best fitting ‘step length’ was six. Then, we used *m* = 6 to build the SVM model and make early warnings.
